# Prediction of MGMT promoter methylation in glioblastoma and grade 4 astrocytoma using fluid-suppressed chemical exchange saturation transfer MRI and machine learning-based segmentation

**DOI:** 10.3389/fonc.2026.1829071

**Published:** 2026-07-23

**Authors:** Tim Salomonsson, Edin Zahirovic, Malte Knutsson, Anina Seidemo, Xavier Saenz Sarda, Patrick Liebig, Stefano Casagranda, Jimmy Lätt, Anna Rydelius, Johan Bengzon, Peter C. M. van Zijl, Linda Knutsson, Pia C. Sundgren

**Affiliations:** 1 Division of Radiology, Department of Clinical Sciences, Lund University, Lund, Sweden; 2 BioMedical Engineering and Imaging Institute, Icahn School of Medicine at Mount Sinai, New York, NY, United States; 3 Division of Pathology, Department of Clinical Sciences, Lund University, Lund, Sweden; 4 Advanced Systems, Magnetic Resonance, Siemens Healthineers AG, Erlangen, Germany; 5 Innovation Lab, Olea Medical, La Ciotat, France; 6 Department of Medical Imaging and Physiology, Skåne University Hospital, Lund, Sweden; 7 Division of Neurology, Department of Clinical Sciences, Lund University, Lund, Sweden; 8 Division of Neurosurgery and Kamprad Laboratory, Department of Clinical Sciences, Lund University, Lund, Sweden; 9 F.M. Kirby Research Center for Functional Brain Imaging, Kennedy Krieger Institute, Baltimore, MD, United States; 10 Department of Radiology, Johns Hopkins University School of Medicine, Baltimore, MD, United States; 11 Department of Neurology, Johns Hopkins University School of Medicine, Baltimore, MD, United States; 12 Department of Medical Radiation Physics, Lund University, Lund, Sweden; 13 Lund University Bioimaging Center (LBIC), Lund University, Lund, Sweden

**Keywords:** amide proton transfer-weighted (APTw) imaging, astrocytoma grade 4, CEST@2ppm, chemical exchange saturation transfer (CEST), glioblastoma, machine learning, magnetic resonance imaging (MRI), MGMT promoter methylation

## Abstract

**Introduction:**

Treatment response in high grade gliomas (HGG) is influenced by O6-methylguanine-DNA methyltransferase promoter methylation (MGMTpm). Chemical exchange saturation transfer (CEST) magnetic resonance imaging (MRI) is a non-invasive approach for potential molecular tumor characterization, although previous studies have reported inconsistent results for MGMTpm. This study investigates fluid-suppressed (FS) CEST metrics and automated tumor segmentation in the preoperative prediction of MGMTpm in HGG.

**Methods:**

3 T MRI including CEST imaging was performed in 44 patients with HGG (mean age 59 years, 16 female, 24 MGMTpm, 39 glioblastoma and five astrocytoma, grade 4). Contrast-enhancing tumor (ET), necrosis, and peritumoral edema were segmented manually and with two machine learning (ML) based models (DeepBraTumIA and Raidionics). Maximum, minimum and percentile-based metrics were extracted from FS amide proton transfer-weighted signal at 3.5 ppm (APTw) and FS CEST signal at 2.0 ppm (CEST@2ppm), normalized with contralateral normal-appearing white matter. The APTw/CEST@2ppm ratio was calculated. Statistical analysis was performed between MGMTpm and non-MGMTpm tumors using group comparisons, Spearman correlation, and receiver operating characteristics with area under curve (AUC), corrected for multiple comparisons.

**Results:**

Significant differences were found in non-MGMTpm relative to MGMTpm tumors: higher 90th percentile and max APTw in necrosis across all segmentation methods; higher 90th percentile APTw, max APTw and max CEST@2ppm in ET with the ML-based models; higher max CEST@2ppm and max ratio in necrosis with Raidionics. The findings with APTw and CEST@2ppm remained consistent in glioblastoma patients and were further supported by significant inverse correlations between the CEST metrics and percentage of MGMTpm. The best-performing parameters for predicting MGMTpm status were the max APTw in ET and necrosis (AUC 0.76–0.82) and max CEST@2ppm in ET and necrosis (AUC 0.67–0.85), achieving higher sensitivity (69–100%) compared to specificity (59–82%).

**Conclusion:**

MGMTpm status in HGG may be predicted with fluid-suppressed CEST MRI. Using publicly available ML-based segmentation tools highlights a potential reproducible workflow in the clinical setting. Future multicenter studies with optimized acquisition protocols and independent, multimodal validation are warranted.

## 1 Introduction

Glioblastoma (GBM), isocitrate dehydrogenase (IDH) wildtype, and astrocytoma, IDH-mutant grade 4, are highly aggressive brain tumors according to the World Health Organization (WHO) classification (1). The molecular profile of the tumor significantly impacts the efficacy of treatment with temozolomide, particularly the methylation status of the O6-methylguanine-DNA methyltransferase (MGMT) promoter (2). The MGMT protein counteracts the DNA alkylation damage caused by temozolomide, leading to resistance to this treatment (2). Methylation of the MGMT gene promoter region leads to decreased expression of MGMT, which correlates with improved survival in GBM and astrocytoma patients, indicating added benefit from temozolomide (3, 4). Early identification of tumor biomarkers therefore plays a crucial role in the management of malignant high-grade gliomas (HGG).

The gold standard to test for MGMT promoter methylation (MGMTpm) involves obtaining tumor tissue and quantitative assessment with methods such as pyrosequencing (5). However, there is additional value in predicting the MGMT status preoperatively. If a tumor is suggested to be non-MGMTpm, this could affect the neurosurgical approach, potentially involving a more aggressive gross total resection to improve survival (6). Magnetic resonance imaging (MRI) has previously been used to predict MGMTpm with varying performance, including conventional, diffusion, and perfusion imaging techniques (7). Molecular imaging using chemical exchange saturation transfer (CEST) MRI is also emerging as a potential non-invasive approach. CEST imaging is based on the principles of chemical exchange of selectively saturated protons, resulting in a measurable decrease in the bulk water magnetization (8, 9). Indirect detection of mobile proteins and peptides via CEST can be achieved using amide proton transfer-weighted (APTw) imaging (8, 9). According to the current CEST consensus guidelines (10), APTw contrast is generated using prolonged saturation with relatively high B_1_ amplitude (~2 µT), ensuring sufficient labeling of amide protons at +3.5 ppm relative to the water resonance frequency. The APTw signal has previously been utilized in glioma grading (11–14), to distinguish tumor recurrence from treatment-related changes (15–17), and to predict overall survival (14, 18). The CEST signal at +2.0 ppm (CEST@2ppm), and the APTw/CEST@2ppm ratio (herein referred to as Ratio) are other metrics that may aid in tumor differentiation (19–23). Moreover, CEST post-processing techniques such as fluid suppression (FS) have been introduced to reduce the signal contribution from fluid-rich compartments such as necrotic or cystic tissues, while retaining the signal in semi-solid tissue compartments (10, 24). FS has previously been used in the distinction of GBM and brain metastases (25), in the molecular profiling of diffuse gliomas (22), and in the assessment of radionecrosis (26).

As summarized in a review article (27), CEST imaging has shown promise in predicting tumor biomarkers such as IDH mutation (18, 22, 28), 1p19q co-deletion (22, 29), p53 overexpression (30) and Ki-67 index (11). However, findings regarding MGMTpm have been inconsistent. Some studies have found higher APTw values in non-MGMTpm tumors using histogram analysis, max APTw% signal or visual assessment (30–33). Conversely, other studies have reported no significant associations between the CEST metrics and MGMTpm (14, 18, 28, 34). To the best of our knowledge, the CEST@2ppm signal, the Ratio, and the utilization of FS have not been used previously in MGMTpm prediction. Notably, some earlier studies may have been hampered by small or skewed sample sizes (31, 32), or the time-consuming task of whole tumor segmentation (28, 30, 33), possibly limiting translation to clinical practice. Automated segmentation of the tumor regions using machine learning (ML) based models may streamline the workflow in larger patient samples and reduce the need for manual delineation (35). Some models that are publicly available for this purpose, and employ a graphical user interface, are DeepBraTumIA (https://www.nitrc.org/projects/deepbratumia) and Raidionics (36). Both models have been evaluated for their clinical utility, especially on preoperative well-demarcated lesions and peritumoral edema (37, 38). The prognostic value of the automatic volumes has also been suggested in survival analyses and longitudinal assessments (39, 40). Some have used the segmentations from DeepBraTumIA and Raidionics to compare volumetric and radiomics-based methods for MGMTpm prediction using conventional MRI (41, 42). These models may therefore have additional benefits in CEST imaging as well, which has not previously been investigated.

Considering the inconclusive body of literature, there is a need for further studies utilizing different CEST contrasts and applying ML-based models in MGMTpm prediction. Therefore, this study aimed to evaluate fluid-suppressed APTw and CEST@2ppm signals, as well as the APTw/CEST@2ppm ratio, to discriminate MGMTpm status in GBM and astrocytoma grade 4, herein collectively referred to as HGG. Segmentation of the contrast-enhancing tumor region, the necrotic core and the peritumoral edema was performed manually and automatically with DeepBraTumIA and Raidionics.

## 2 Materials and methods

### 2.1 Patients and pathology

This study included 44 patients diagnosed with HGG (39 GBM and five astrocytoma grade 4), according to the WHO 2021 classification (1). The subjects were included retrospectively as part of an ongoing longitudinal single-center cohort study. Inclusion criteria were: age ≥18 years old, prior imaging indicating a suspected brain tumor, and biopsy or surgery for histopathological analysis. Exclusion criteria were: any contraindication to performing MRI, such as incompatible pacemaker or metallic implants, only post-operative MRI, missing data, and being unable to sign informed consent. Some of the patients were diagnosed according to the earlier WHO 2016 classification; these cases were retrospectively re-classified according to WHO 2021 by a senior neuropathologist (XSS). MGMT status was routinely analyzed with pyrosequencing, using a cut-off > 9% to define MGMTpm (43). The study was conducted in accordance with the Declaration of Helsinki and approved by the Regional Ethics Board in Lund, Sweden (#2016/531, #2017/866, #2018/245, #2018/993) and subsequently by the Swedish Ethical Review Authority (#2019-03569, #2020-00851, #2021-06311-02). All patients signed written informed consent, and neither inclusion nor exclusion altered the management of the patients.

### 2.2 Image acquisition

The patients were examined on a 3 T MRI scanner with a 20-channel head coil (MAGNETOM Prisma, Siemens Healthcare, Erlangen, Germany). The conventional sequences included: T1-weighted magnetization-prepared rapid gradient echo (MPRAGE) before and after gadolinium contrast administration with a repetition time (TR) = 1900 ms, echo time (TE) = 2.54 ms, inversion time (TI) = 900 ms, flip angle (FA) = 9°, axial resolution = 1x1 mm^2^, slice thickness = 1 mm and matrix size = 256x256x192; T2-weighted fluid-attenuated inversion recovery (FLAIR) with TR = 5000 ms, TE = 393 ms, TI = 1800 ms, FA = 120°, axial resolution = 1x1 mm^2^, slice thickness = 1 mm and matrix size = 256x256x176; and a T2-weighted turbo spin echo sequence with TR = 6000 ms, TE = 100 ms, FA = 150°, axial resolution = 0.43x0.43 mm^2^, slice thickness = 6 mm and matrix size = 448x512x27.

CEST imaging was performed using a vendor-specific gradient echo APTw research sequence with the following parameters: TR = 10 ms, TE = 2.54 ms, FA = 12°, axial resolution = 2x2 mm^2^, slice thickness = 4 mm and matrix size = 100x128x22. The saturation module consisted of five hyperbolic secant radiofrequency pulses with B_1_ = 2.0 μT, pulse duration = 100 ms and interpulse delay = 61 ms. This resulted in a total saturation time (T_sat_) = 744 ms and a duty cycle (DC) = 67%. The pulses were applied at 21 equally spaced frequency offsets ranging from -5 ppm to 5 ppm relative to the bulk water resonance frequency, obtaining the Z-spectrum. An unsaturated reference image (S_0_) was obtained at -150 ppm. The CEST sequence was performed before contrast agent administration as part of the imaging protocol.

### 2.3 Post-processing

The CEST MRI data was post-processed with Olea Sphere (version 3.0.36, Olea Medical, La Ciotat, France), using the automatic workflow within the program, as follows. All images underwent temporal denoising (44), spatial denoising (45) and rigid-body motion correction (46). The voxel-wise water signal intensity (S_sat_) was normalized to S_0_ for each saturation frequency offset (Δω). Following normalization, B_0_ correction was performed by shifting the minimum of the direct water saturation signal in each voxel to 0 ppm (47). The APTw% signal was then calculated using linear interpolation of the Z-spectrum (48), using the difference between the integrals -3.5 ± 0.5 ppm and 3.5 ± 0.5 ppm as described in (25). To address fluid signal contributions, FS was applied to the processed APTw% images according to Schure et al. using a target tissue spillover dilution factor (σ′_target_) set to 0.35^2^ (24). This correction reduces the signal contributions from fluid-rich compartments with minimal effect on the APTw% signal in semi-solid tissue from normal appearing white matter (NAWM) (24). Additionally, since spillover-corrected FS is a scaling procedure performed during post-processing that retains the Z-spectral intensities of NAWM, no substantial alterations occur in the magnetization transfer background in the tumor voxels. Also, contrary to acquisition-based methods, scaling does not affect the signal-to-noise properties of the data (24). It affects contrast because unwanted signals are suppressed, but relevant signals are maintained, keeping the APTw% signal in tumor comparable to that without suppression (23, 24). The CEST@2ppm% signal was calculated using the same method of linear interpolation with the difference between the integrals -2.0 ± 0.5 ppm and 2.0 ± 0.5 ppm. FS is also applicable to other frequency offsets (24), and the same approach was therefore applied to the CEST@2ppm% images, but with σ′_target_ = 0.25^2^ reflecting the closer proximity to direct water saturation (23). The FS APTw% and FS CEST@2ppm% images were then used to calculate the APTw/CEST@2ppm ratio. Lastly, the CEST images underwent rigid co-registration to the post-contrast T1-weighted MPRAGE (46).

### 2.4 Segmentation

Segmentation was performed using the conventional sequences (T1-weighted MPRAGE pre- and post-contrast, FLAIR and T2-weighted) manually in 3D Slicer (version 5.8.1, https://www.slicer.org) (49), as well as automatically using DeepBraTumIA (version 2.1.0, https://www.nitrc.org/projects/deepbratumia) and Raidionics (version 1.3.1, https://github.com/raidionics/Raidionics) (36). The images were first processed through DeepBraTumIA, performing rigid co-registration to the T1-weighted post-contrast image, using the MNI152 atlas, 1 mm^3^ isotropic voxel size and matrix size 182x218x182 (50). Skull-stripping was thereafter performed using the HD Brain Extraction Tool (51). Automatic segmentation was subsequently carried out using the pre-operative segmentation modules in DeepBraTumIA and Raidionics, respectively. Additionally, manual segmentation was performed in 3D Slicer by two junior researchers (EZ and TS) under the guidance of a senior board certified neuroradiologist (PCS). The segmentations generated from DeepBraTumIA and Raidionics were imported into 3D Slicer and applied to the co-registered CEST images.

**Figure 1:**
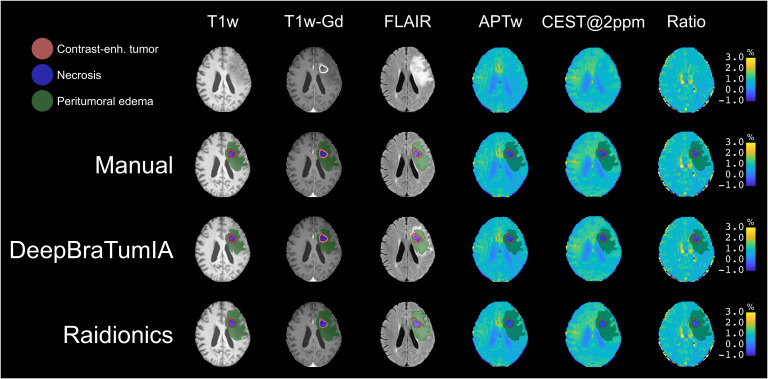
A single slice of the whole tumor segmentations performed in a 62-year-old patient with glioblastoma, methylated MGMT promoter. Images include conventional T1-weighted MRI before (T1w) and after (T1w-Gd) contrast administration, fluid-attenuated inversion recovery (FLAIR), fluid-suppressed amide proton transfer weighted map at 3.5 ppm (APTw), fluid-suppressed CEST signal map at 2.0 ppm (CEST@2ppm) and the APTw/CEST@2ppm ratio. The contrast-enhancing tumor (red), necrosis (blue) and peritumoral edema (green) are segmented manually and automatically with DeepBraTumIA and Raidionics.

**Figure 2:**
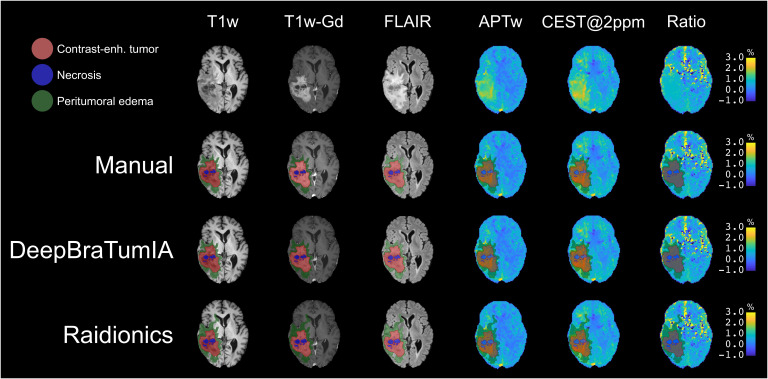
A single slice of the whole tumor segmentations performed in a 61-year-old patient with glioblastoma, non-methylated MGMT promoter. Images include conventional T1-weighted MRI before (T1w) and after (T1w-Gd) contrast administration, fluid-attenuated inversion recovery (FLAIR), fluid-suppressed amide proton transfer weighted map at 3.5 ppm (APTw), fluid-suppressed CEST signal map at 2.0 ppm (CEST@2ppm) and the APTw/CEST@2ppm ratio. The contrast-enhancing tumor (red), necrosis (blue) and peritumoral edema (green) are segmented manually and automatically with DeepBraTumIA and Raidionics.

The three segmentation methods generated volumes of interest (VOIs) encompassing the entire contrast-enhancing tumor (ET), necrotic area and peritumoral edema, respectively. **Figures 1**, **2** show a single slice of the VOIs in two representative cases of an MGMTpm and a non-MGMTpm tumor. The minimum (min), maximum (max), median, 10th and 90th percentile of the FS APTw%, FS CEST@2ppm% and the APTw/CEST@2ppm ratio were systematically extracted from all the VOIs using a custom Python script in 3D Slicer. An additional circular VOI was manually placed in NAWM across 6 adjacent slices in the contralateral lobe at the level of the tumor center, with a total average size of 1200 mm^3^. The metrics were then divided with the corresponding median value in NAWM to calculate the normalized FS APTw% (nAPTw%) and normalized FS CEST@2ppm% (nCEST@2ppm%).

Throughout the segmentation process, the authors (EZ and TS) were blinded to MGMT-status and to the results of the other ML-based models.

### 2.5 Statistics

All statistical analyses were conducted using IBM SPSS (version 29.0.20, IBM Corp, Armonk, NY, United States). The manually segmented VOIs and the automatic output from DeepBraTumIA and Raidionics were evaluated separately. The Shapiro-Wilk test was performed to assess normality. Depending on the outcome, an independent samples t-test or Mann-Whitney U test was chosen to determine differences in CEST metrics (nAPTw%, nCEST@2ppm%, APTw/CEST@2ppm ratio) between MGMTpm and non-MGMTpm tumors. Spearman correlation was performed to assess the relationship between the percentage of MGMTpm (MGMTpm%) and the CEST metrics that were significant in the group comparison. Receiver operating characteristic (ROC) curves evaluated the predictive ability of the above-mentioned significant CEST metrics, including area under curve (AUC), sensitivity and specificity at Youden’s optimal cut-off. Regarding patient characteristics, the t-test or Mann-Whitney U test was performed for continuous variables, and the Chi-square test or Fisher’s exact test for categorical variables. Each analysis was False Discovery Rate (FDR) corrected for multiple comparisons, with adjusted p < 0.05 defined as statistically significant. The FDR corrected p-values are presented in **Supplementary Tables 1**–**3** and the uncorrected p-values in **Supplementary Tables 4**–**7**. All the analyses were performed both in the entire cohort and only in patients with GBM.

## 3 Results

### 3.1 Patient characteristics

Patient characteristics are summarized in **Table 1**. Among the 44 patients (mean age 59 years, 16 female), 24 had MGMTpm tumors. Of the 39 patients with GBM, 20 were MGMTpm. There were no significant differences in age (p = 0.72), sex (p = 0.42), tumor type (p = 0.22), location (p = 0.31), laterality (p = 0.47) or type of surgery (p = 0.28) within the two MGMT groups. No significant differences were seen in the other biomarkers that are presented in **Table 1**. There were also no differences between MGMTpm and non-MGMTpm tumors in the median NAWM values used for normalization (data not shown).

**Table 1:** Patient characteristics, split by MGMT promoter methylation (MGMTpm) status.

Variable	MGMTpm	non-MGMTpm	Total
Total patients, n (%)	24 (55%)	20 (45%)	44 (100%)
Age [years], mean (SD)	59.2 (13.2)	57.6 (14.9)	58.5 (13.9)
Sex, n (%)			
Male	14 (58%)	14 (70%)	28 (64%)
Female	10 (42%)	6 (30%)	16 (36%)
Tumor type, n (%)			
Glioblastoma, grade 4	20 (83%)	19 (95%)	39 (89%)
Astrocytoma, grade 4	4 (17%)	1 (5%)	5 (11%)
Biomarkers			
IDH1 mutation, n (%)	4 (17%)	1 (5%)	5 (11%)
p53 overexpression, n (%)	10 (42%)	3 (15%)	13 (30%)
ATRX-wildtype, n (%)	21 (88%)	13 (65%)	34 (77%)
Ki-67 index [%], median (IQR)	20 (22)	14 (13)	17 (17)
Tumor laterality, n (%)			
Left	11 (46%)	10 (50%)	21 (48%)
Right	9 (38%)	9 (45%)	18 (41%)
Midline	4 (17%)	1 (5%)	5 (11%)
Tumor location, n (%)			
Frontal	7 (29%)	4 (20%)	11 (25%)
Temporal	4 (17%)	8 (40%)	12 (27%)
Occipital	3 (13%)	4 (20%)	7 (16%)
Parietal	3 (13%)	1 (5%)	4 (9%)
Midline	4 (17%)	1 (5%)	5 (11%)
Insula	1 (4%)	2 (10%)	3 (7%)
Basal ganglia	2 (8%)	0 (0%)	2 (5%)
Type of surgery, n (%)			
Biopsy	12 (50%)	6 (30%)	18 (41%)
Partial resection	8 (33%)	7 (35%)	15 (34%)
Gross total resection	4 (17%)	7 (35%)	11 (25%)

MGMT, O6-methylguanine-DNA methyltransferase. MGMTpm, MGMT promoter methylation. CEST, Chemical exchange saturation transfer. n, Number of patients. SD, Standard deviation. IQR, Interquartile range. IDH1, Isocitrate dehydrogenase 1. p53, Tumor protein p53. ATRX, Alpha thalassemia/mental retardation syndrome X-linked. Ki-67, Antigen Kiel 67.

### 3.2 CEST metrics

**Figure 3:**
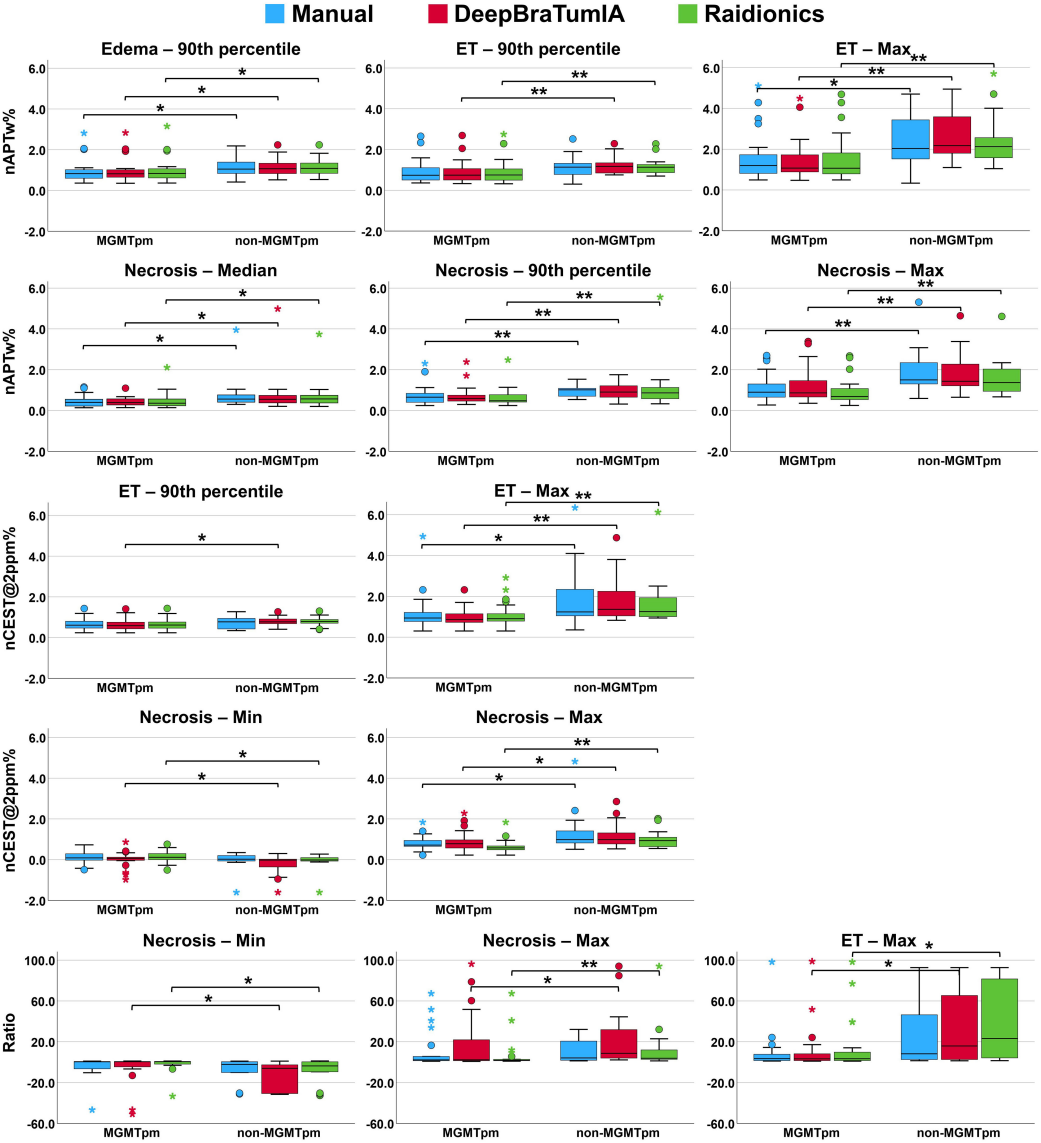
Boxplots comparing MGMTpm and non-MGMTpm results for nAPTw%, nCEST@2ppm%, and APTw/CEST@2ppm ratio in contrast-enhancing tumor (ET), necrosis and edema. The plots are colored according to the segmentation method (Manual, DeepBraTumIA, Raidionics). The boxes represent the median and interquartile range (IQR), and the whiskers represent the minimum and maximum values that are not outliers (defined as 1.5 x IQR below the first quartile or above the third quartile, denoted with a colored circle) or extreme outliers (more than 3.0 x IQR, denoted with a colored asterisk). Differences between MGMT promoter methylated (MGMTpm) and non-methylated (non-MGMTpm) tumors are noted with a connecting line and a black asterisk if the results were significant before (*) or after (**) correction for multiple comparisons.

A summary of the CEST metrics obtained from the three segmentation methods is presented in **Figure 3** and **Supplementary Table 1**. After FDR correction, patients with non-MGMTpm displayed significantly higher 90th percentile and max nAPTw% in necrosis across all segmentation methods, and higher 90th percentile and max nAPTw% in ET with the ML-based models (DeepBraTumIA and Raidionics), compared to patients with MGMTpm. Significantly higher max nCEST@2ppm% in ET with the ML-based models and higher max nCEST@2ppm% in necrosis, segmented with Raidionics, were also seen in non-MGMTpm compared to MGMTpm tumors. Non-MGMTpm tumors also showed significantly higher max Ratio in necrosis when segmented with Raidionics. The results were consistent when analyzing only patients with GBM (**Supplementary Table 2**), except for the findings using the Ratio that became non-significant after correction for multiple comparisons.

**Figure 4:**
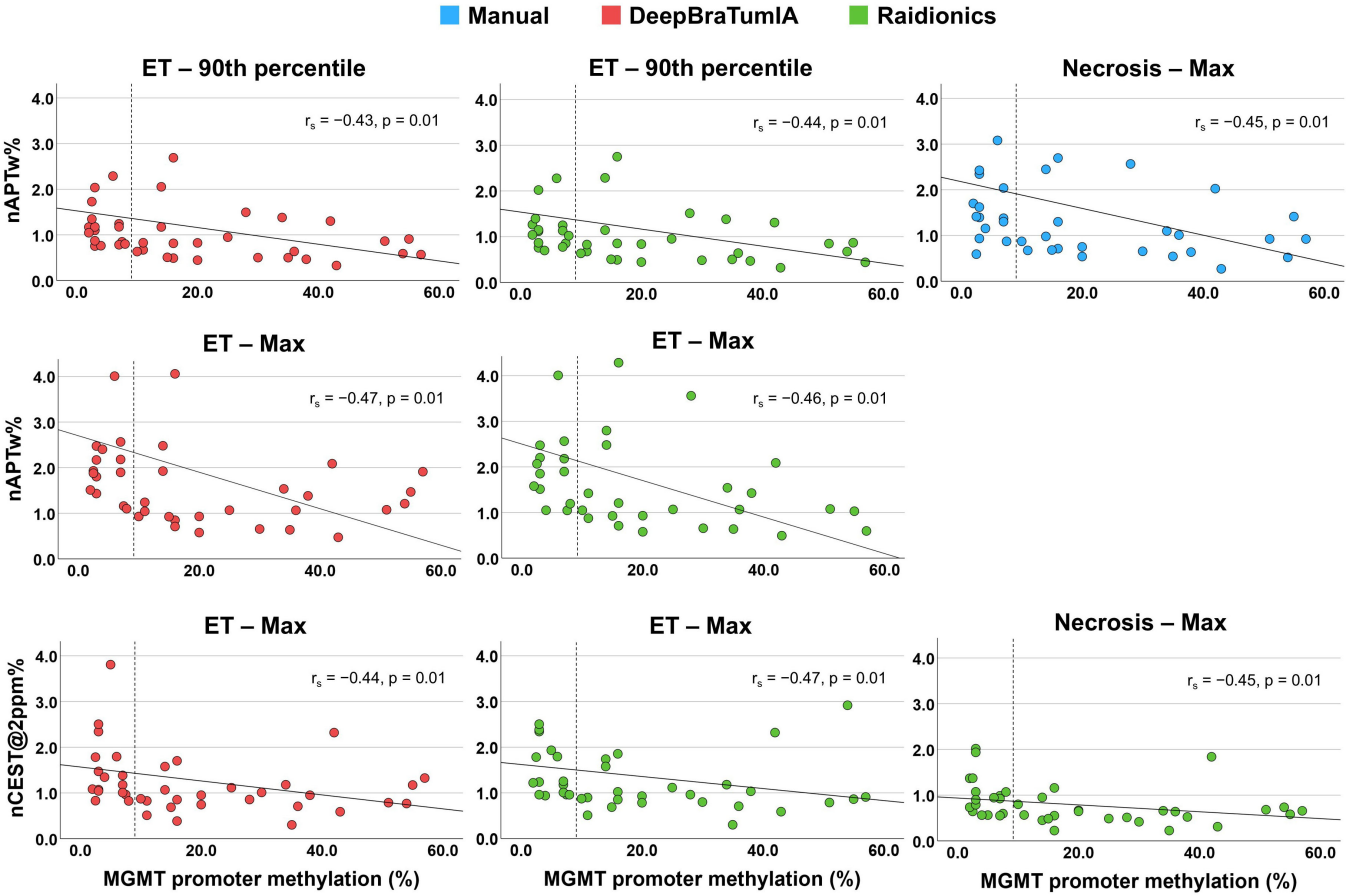
Scatterplots displaying the distribution of nAPTw% and nCEST@2ppm% as a function of the percentage of MGMT promoter methylation, with the metrics in contrast-enhancing tumor (ET) and necrosis that showed the strongest significant correlations. The plots are colored according to the segmentation method (Manual, DeepBraTumIA, Raidionics). The dashed line represents the clinical cut-off > 9% to define MGMTpm. Spearman’s rank correlation coefficient (r_s_) and p-values corrected for multiple comparisons are presented for each metric.

Further investigation of the significant parameters with Spearman correlation as a function of MGMTpm percentage is shown in **Figure 4** and **Supplementary Table 3**. After FDR correction, there were significant inverse correlations between MGMTpm% and the 90th percentile and max nAPTw% in ET and necrosis, and the max nCEST@2ppm% in ET and necrosis. The MGMTpm% also inversely correlated with the max Ratio in necrosis.

**Figure 5:**
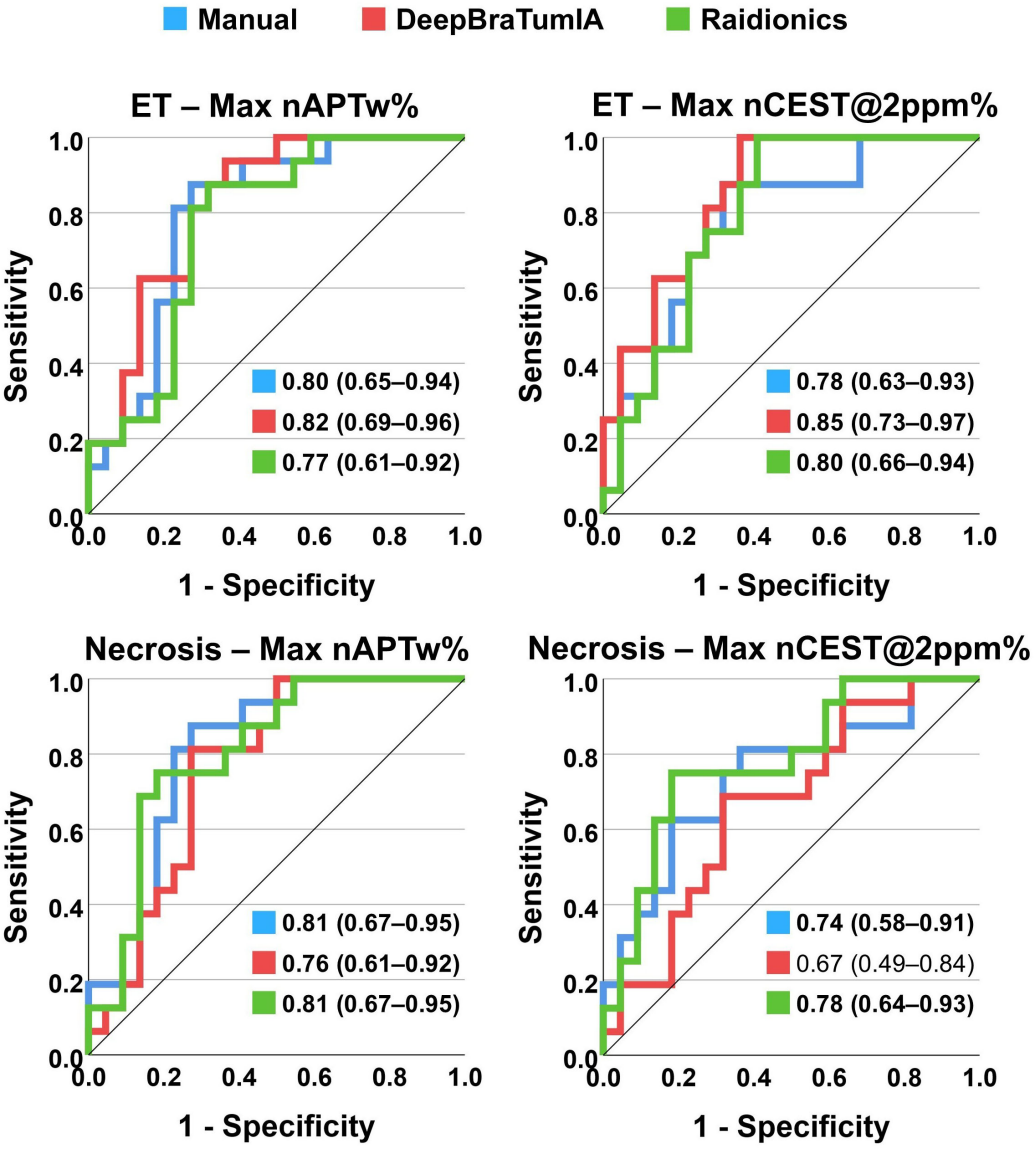
Receiver operating characteristic (ROC) curves representing the best nAPTw% and nCEST@2ppm% metrics in distinguishing between MGMT promoter methylated and non-methylated tumors. The segmentation methods (Manual, DeepBraTumIA, Raidionics) are color coded and presented in contrast-enhancing tumor (ET) and necrosis. The area under curve (AUC) with 95% confidence interval is presented for each metric, with significant metrics after correction for multiple comparisons highlighted in bold.

A summary of the ROC analyses after FDR correction is presented in **Figure 5** and **Supplementary Table 3**. Significant ROC curves were seen in the 90th percentile and max nAPTw% in ET and necrosis. The ROC analyses were also significant in the max nCEST@2ppm% in ET and necrosis, and the max Ratio in necrosis. The best parameter for each VOI was the max nAPTw% in ET (AUC 0.77–0.82, sensitivity 88–94%, specificity 64–73%, at cut-off 1.41–1.50), the max nAPTw% in necrosis (AUC 0.76–0.81, sensitivity 75–88%, specificity 73–82%, at cut-off 1.13–1.20), the max nCEST@2ppm% in ET (AUC 0.78–0.85, sensitivity 88–100%, specificity 59–68%, at cut-off 0.93–1.03) and the max nCEST@2ppm% in necrosis (AUC 0.67–0.78, sensitivity 69–81%, specificity 64–82%, at cut-off 0.73–0.91). The ROC curves of these parameters are summarized in **Figure 5**.

## 4 Discussion

This study evaluated the diagnostic performance of the normalized fluid-suppressed CEST signals at 3.5 ppm and 2.0 ppm for differentiating MGMT status in HGG. APTw imaging is commonly studied in gliomas (10), and CEST@2ppm is also emerging as a novel marker of brain tumor differentiation (21, 23). The CEST@2ppm signal and the ratio has previously been used in the prediction of IDH mutation and 1p19q co-deletion (22, 29). However, to the best of the authors’ knowledge, no previous study has utilized both APTw, CEST@2ppm and their ratio for MGMTpm prediction, warranting an exploratory comparison of their performance. Other key novelties of this study in the context of CEST MRI include treating MGMTpm as both a binary and continuous variable and using both manual and automatic segmentation methods. After correction for multiple comparisons, our results indicate that non-MGMTpm HGG display higher max and 90th percentile nAPTw% and nCEST@2ppm% in contrast-enhancing tumor and central necrosis. Interestingly, few significant findings were seen when using the APTw/CEST@2ppm ratio, with higher variability and lower diagnostic accuracy. This may be a consequence of the reduced contrast to noise, suggesting that the Ratio may not be equally suited in distinguishing MGMTpm compared to other biomarkers such as IDH and 1p19q status (22). Our results are in agreement with an earlier study that investigated multi-pool CEST signals using Lorentzian fitting, which identified that the signal at 3.5 ppm was predictive of MGMTpm status, with the 90th percentile showing the highest accuracy (33). Some other studies have also found either the max or 90th percentile APTw% values to be higher in non-MGMTpm tumors (30, 31). The exclusion of outliers by using the 90th percentile may therefore further enhance the differences in some cases. Conversely, our results disagree with some other studies that were unable to distinguish MGMTpm status using the same metrics (14, 18, 28, 34). Compared to APTw maps computed at 3.5 ppm, the signal derived at 2.0 ppm has been studied less frequently and cannot be attributed to a singular metabolite (52). The main signal contributions are suggested to be guanidinium protons in arginine side chains in mobile proteins, creatine and phosphocreatine, and possibly to a lesser extent contamination by the glutamate pool (52–54). An elevated CEST@2ppm signal could therefore be seen if there is a relative increase in mobile proteins with arginine residues, compared to the decrease in creatine signal seen in brain tumors (19, 21, 52, 54). While the CEST sequence performed in our study was mainly weighted towards amides at 3.5 ppm, a previous publication suggests that similar settings (B_1_ = 1.7 μT, T_sat_ = 0.9 s) may also be used to generate the 2.0 ppm signal intensity peak at 3 T (55). Some clinical studies have also been performed at 3 T with comparable parameters to ours (22, 23, 29). Given the diversity in methodology and in earlier results, further research is needed to optimize the use of APTw and CEST@2ppm in MGMTpm prediction.

Additionally, to the best of our knowledge, we are the first to evaluate the correlation between the extent of MGMTpm% and the obtained CEST values. The clinical local routine is a cut-off > 9% to define MGMTpm, in line with a previous Cochrane review (43). However, some studies suggest a continuous association between the percentage of methylated CpG sites and survival as well as volumetric outcomes in HGG (56, 57). In our study, the strongest significant correlations were seen between MGMTpm% and the max nAPTw% and nCEST@2ppm% values in ET and necrosis. This may indicate an inverse association with higher CEST metrics at lower degrees of methylation, consistent with the binary classification. Interestingly, this relationship may not be perfectly linear due to the moderately strong correlation coefficients, up to −0.48. Other ways of stratification based on MGMTpm% should therefore be explored if there instead is a non-linear relationship, as suggested from a prognostic point of view in some previous studies (58, 59). Regardless, the MGMTpm status itself should not be influenced by the composition of contrast-enhancing or necrotic tissue, particularly when there is a high tumor cellularity (60). Therefore, an elevated signal in both tumor- and necrotic-appearing regions on MRI would be expected when FS has been applied, correcting for the non-specific fluid and diluted protein pool (24).

The ability to preoperatively predict MGMTpm status has significant clinical implications regarding the neurosurgical approach and subsequent management (6). Especially in patients where the value of surgery is low (such as low performance status, older age, tumor location, and larger tumor volume) (61), being able to distinguish tumor markers could have a substantial impact on the choice and the level of oncological therapy. Therefore, both IDH-wildtype and IDH-mutant HGG were included to reflect the clinical setting when histopathological information is unknown prior to operation. The best predictive ability with high AUC was seen using the max nAPTw% and nCEST@2ppm% values in ET and necrosis. Generally, higher sensitivity than specificity was noted when using Youden’s index, generating lower cut-off values. This suggests that there is a high accuracy in capturing all non-MGMTpm tumors with an elevated CEST signal, i.e. tumors benefitting from a more aggressive neurosurgical approach, regardless of IDH-status (6). Additionally, in cases of clearly eloquently located tumors with a situation of limited access to tumor tissue by surgery, the method may aid oncological decision making. When interpreting the findings in the present study, potential biological confounders between GBM and high-grade astrocytoma should be considered. Notably, IDH mutations may lead to the accumulation of 2-hydroxyglutarate and downstream alterations of other metabolites, such as a reduction of glutamate and glutamine observed with MR spectroscopy (62). This could lead to lower CEST signal intensities in IDH-mutant tumors compared to GBM (18, 22, 28), especially due to the reduced contribution of glutamate and amine protons in the 2–3 ppm range (52, 53). To address this confounder, the analyses were also performed in only patients with GBM, and the significant results remained consistent overall with APTw and CEST@2ppm, as shown in **Supplementary Table 2**.

Alternatively, our findings may reflect several other differences in the tumor microenvironment (TME) depending on the phenotype. Tumor cells alter several cellular processes (such as a shift towards aerobic glycolysis with increased production and excretion of lactic acid) causing a pH gradient with an acidic extracellular pH and relatively alkaline intracellular pH (63, 64). These changes may further promote pro-tumorigenic processes such as neoangiogenesis, proliferation, infiltration, evasion of apoptosis and resistance to treatment, leading to decreased survival in non-MGMTpm tumors (3, 4, 63). The more aggressive features of non-MGMTpm tumors can also be captured as an increase in relative cerebral blood volume in the tumor region due to microvascular remodeling and reduced apparent diffusion coefficient associated with tumor cell proliferation (7, 65). Neoangiogenesis with high mobile protein content, increased cellularity and an elevated intracellular pH in this subgroup could therefore be contributing factors to our findings (7, 10, 52, 66). While the same results could be expected in the peritumoral region due to the diffusely infiltrating nature of glioma cells (67), the differences in the edema segments were non-significant after FDR correction in our study. This warrants additional methodological considerations, such as the investigation of separate subregions to possibly distinguish different contributing causes of FLAIR hyperintensity such as tumor infiltration or vasogenic edema (67). Furthermore, MGMT expression in itself may also play a role in shaping the TME. Some studies suggest that an unmethylated MGMT promoter (leading to upregulation of MGMT) may lead to an immunogenetically active TME with increased presence of macrophages, CD4+ and CD8+ T-cells (68). When activated, these cells increase their glycolytic metabolism contributing to extracellular lactic acid accumulation and a similar pH gradient as the tumor cells (69). A higher proportion of tumor-associated macrophages, especially in the presence of lactic acid, will then be polarized toward the immunosuppressive M2 phenotype (70). These cells will further promote tumor growth through several signaling mechanisms, cytokines and recruitment of regulatory T cells, inhibiting the other immune cells in the TME such as natural killer cells and dendritic cells (70). Conversely, low MGMT expression (with high MGMTpm) may instead promote an inflammatory environment with higher infiltration of cytotoxic CD8+ T-cells (68). This, in turn, could instead lead to less alkaline intracellular pH, causing apoptosis and decreased tumor cell burden, resulting in low CEST metrics in the MGMTpm group (69). Future studies should therefore incorporate a multimodal approach with advanced imaging, histopathology, molecular and immunological analyses to validate our findings with regards to MGMTpm status and the TME.

In addition, our results add to the growing body of literature that evaluates ML-based tools in neurooncology (35). To the best of our knowledge, this is the first study to assess publicly available segmentation models in MGMT prediction with CEST MRI. In this work, the models were applied to conventional MRI sequences, and the resulting segmentations were subsequently transferred to the co-registered CEST images for quantitative analysis. The highest diagnostic accuracy was observed when using segmentations generated by DeepBraTumIA, although the differences in AUC between methods were minor. Given the potential inter-reader variability in brain tumor segmentation between radiologists, utilizing ML-based models may facilitate both reproducibility in research and streamlining of the workflow in a clinical setting (35). Compared to traditional manual placement of two-dimensional regions of interest, automatic methods have the advantage of performing efficient VOI-based sampling, encompassing multiple image slices across the whole tumor. This may be especially important for feature extraction on images with lower resolution and accounting for regional tumor heterogeneity (71). DeepBraTumIA and Raidionics have already shown promise when using volumetric data and radiomics from conventional images for prediction of MGMTpm (41, 42). This study demonstrates the value in using these tools to aid in the analysis of CEST metrics. Continued validation against manual delineation is warranted, due to potential bias within each model’s training data and a risk of under- or over-sampling particularly in diffuse tumors with complex morphology (35, 37, 38). Notably, the precision of the ML models has been evaluated in a previous study which included a subgroup of the same patients (n = 30) (42). Compared to manual segmentation, DeepBraTumIA and Raidionics showed high intraclass correlation coefficients and accuracy in both ET and edema (0.90–0.96 and 80–91%), and higher variability in necrosis (0.50 and 67–80%) (42). This indicates robustness and high spatial overlap when automatically defining the tumor and peritumoral edema in these patients. The central hypointense lesion on T1-weighted images is often the smallest VOI, and minor differences between methods could lead to lower perceived accuracy of necrosis segmentation especially on conventional MRI (42). However, when applied to lower resolution CEST images, these variations may not result in a substantial discordance of the calculated metrics. Future research may therefore benefit from a combined approach, where automated segmentations are reviewed and minimally corrected by expert readers to account for potential internal bias within the models (35).

This study is not without limitations. While the overall cohort was larger compared to some previous clinical CEST studies (28, 30–32, 34), the sample size and single-center design lead to reduced generalizability of the results. Although the patients were originally recruited prospectively before confirmed diagnosis, the inclusion and analyses performed in this study were retrospective. Due to the limited number of previous papers on MGMTpm prediction and application of ML-based segmentation models in CEST MRI, the design was considered exploratory and hypothesis-generating. In this context, the current findings provide a basis for further research rather than definitive clinical validation. Nevertheless, the lack of an independent validation cohort is a limitation with this approach. Therefore, the findings should be interpreted with caution, and the robustness needs to be confirmed in larger, preferably prospective multi-center studies. Additional limitations relate to the CEST acquisition and post-processing. Due to hardware constraints, the CEST sequence was performed with a shorter saturation time compared to the current consensus recommendations at 3 T (10). This leads to increased contributions from mobile protein containing blood vessels and decreased contribution from semi-solid tissue (10). The lower spatial resolution of the CEST images compared to conventional MRI may also lead to partial volume effects during co-registration and application of the segmentations, potentially introducing variability in the extracted metrics. However, similar limitations have been common in clinical CEST studies and have not previously hindered the detection of biologically relevant signal differences (27). To ensure validity, future studies should attempt to reproduce the current results with optimized parameters according to the consensus recommendations and clinically approved sequences when applicable (10). The approach of using the integral over a range of frequency offsets when calculating the CEST images may also introduce signal contributions from other metabolites with nearby resonance frequencies (10, 52). However, previous research suggests that the method chosen in this study instead reduces the influence of local B_0_ inhomogeneities (48). Other methods of performing CEST analysis include the magnetization transfer ratio asymmetry (MTR_asym_) analysis at 3.5 ppm and 2.0 ppm (10). A comparison between the performance of the integral approach and the MTR_asym_ analysis may therefore be warranted in the future. Regarding B_0_ inhomogeneity correction, we used information derived directly from the acquired Z-spectrum to perform the frequency alignment. More rigorous approaches for B_0_ mapping are available, particularly those based on dedicated saturation-based techniques such as WASABI (72) or WASSR (73), which allow B_0_ estimation without contamination from the CEST spectral signal. These methods could be considered to further improve robustness, although cross-vendor standardization remains a key challenge (74). Additionally, no B_1_ correction was applied in the present analysis. Incorporating B_1_ mapping and correction tailored for APTw imaging (75) in future work may improve quantitative consistency, particularly in frontal brain regions where reduced B_1_ efficiency can affect saturation transfer measurements. Fluid suppression, although theoretically motivated, may still in select cases impact the signal intensity in both healthy and pathological tissue, affecting the values in NAWM (23, 24). The correction factors chosen for the APTw and CEST@2ppm images were therefore consistent with previous literature, maintaining reproducibility (23, 24, 26, 76). Future studies could explore other methods of FS (76, 77), as well as patient-specific correction factors in order to improve the accuracy of MGMTpm prediction. Lastly, the current ML models’ labeling of edema encompasses the whole peritumoral FLAIR hyperintensity, which may include both vasogenic edema and tumor infiltration. Therefore, future studies and models may benefit from a multiparametric approach, separating the FLAIR hyperintensity into subregions and utilizing different advanced MRI sequences (67).

In summary, several methodological challenges in acquisition and post-processing strategies remain to be further explored, limiting generalizability at this stage. Based on our findings, important aspects to consider when facilitating broader reproducible clinical adoption include: utilizing a CEST sequence with optimized saturation parameters (preferably B_1_ = 2.0 μT, T_sat_ = 2 s, DC ≥ 90%) (10); ensuring technical quality with adequate B_0_ and B_1_ correction independently of vendor (72–75); implementing necessary post-processing steps including denoising, motion correction, MTR_asym_ calculation and fluid suppression to enhance visibility (10, 24, 44–46, 76–78); when feasible, performing semi-automatic whole tumor segmentation with minimal manual correction for accurate feature extraction (35, 71); defining relevant quantitative metrics when evaluating the APTw and CEST@2ppm signals, where percentile-based metrics may be more robust to outliers compared to maximum values (31, 33). These considerations highlight the importance of a standardized protocol to ensure reproducibility in future research and clinical work with CEST MRI.

## 5 Conclusion

This study utilized manual and ML-based segmentations to extract the fluid-suppressed APTw signal at 3.5 ppm, the CEST signal at 2.0 ppm, and calculated the APTw/CEST@2ppm ratio to predict MGMTpm status in HGG. Our results highlight the additional value of CEST imaging in preoperative MGMTpm prediction, which potentially could be used as an additional tool to aid neurosurgical and neurooncological decision making. The usage of ML-based models may further streamline the workflow. The findings should be validated in larger independent cohorts with optimized sequence parameters, as well as comparing different imaging modalities and post-processing methods to generate a feasible framework in future clinical practice.

## Data Availability

The raw data supporting the conclusions of this article will be made available by the authors, without undue reservation.
