# Supplementary material for: Prediction of MGMT promoter methylation in glioblastoma and grade 4 astrocytoma using fluid-suppressed chemical exchange saturation transfer MRI and machine learning-based segmentation
Source: Front Oncol. 2026 Jul 23;16:1829071. doi: 10.3389/fonc.2026.1829071 (PMC13441775; doi:10.3389/fonc.2026.1829071)
Supplement: Supplementary file 1 [file Supplementaryfile1.docx]

Supplementary Material

**Supplementary Table 1.** Summary of the normalized APTw% (nAPTw%), CEST@2ppm% (nCEST@2ppm%) and their Ratio from the three segmentation methods comparing MGMT promoter methylated (MGMTpm) and non-methylated (non-MGMTpm) tumors in the whole cohort (n = 44). The data was nonparametric; therefore, the median values and interquartile range (IQR) are presented for each variable. P-values after correction for multiple comparisons are presented, with significant differences in bold.

|  | |  | **nAPTw%** | | | | | **nCEST@2ppm%** | | | | |
| --- | --- | --- | --- | --- | --- | --- | --- | --- | --- | --- | --- | --- |
| **Segment** | **Parameter and method** | | **MGMTpm** | | **non-MGMTpm** | |  | **MGMTpm** | | **non-MGMTpm** | |  |
|  |  |  | **Median** | **IQR** | **Median** | **IQR** | **P-value** | **Median** | **IQR** | **Median** | **IQR** | **P-value** |
| **ET** | **Min**  Manual  DeepBraTumIA  Raidionics | | 0.04  0.10  0.06 | 0.31  0.40  0.29 | 0.10  -0.12  -0.09 | 0.55  0.82  0.61 | 0.51  0.11  0.24 | -0.001  0.04  0.04 | 0.43  0.36  0.39 | -0.02  -0.02  -0.14 | 0.37  0.47  0.46 | 0.74  0.44  0.16 |
|  | **10th percentile**  Manual  DeepBraTumIA  Raidionics | | 0.30  0.29  0.29 | 0.36  0.36  0.32 | 0.40  0.40  0.43 | 0.34  0.36  0.37 | 0.24  0.40  0.29 | 0.26  0.26  0.24 | 0.31  0.29  0.30 | 0.22  0.22  0.20 | 0.26  0.27  0.27 | 0.88  1.00  1.00 |
|  | **Median**  Manual  DeepBraTumIA  Raidionics | | 0.52  0.47  0.50 | 0.43  0.42  0.41 | 0.67  0.71  0.71 | 0.46  0.44  0.47 | 0.16  0.09  0.16 | 0.41  0.41  0.40 | 0.31  0.31  0.31 | 0.42  0.43  0.43 | 0.31  0.32  0.34 | 0.86  0.81  0.86 |
|  | **90th percentile**  Manual  DeepBraTumIA  Raidionics | | 0.74  **0.74**  **0.75** | 0.64  **0.61**  **0.60** | 1.13  **1.17**  **1.13** | 0.56  **0.61**  **0.47** | 0.13  **0.03**  **0.03** | 0.61  0.59  0.62 | 0.35  0.33  0.31 | 0.78  0.78  0.80 | 0.52  0.26  0.28 | 0.54  0.12  0.16 |
|  | **Max**  Manual  DeepBraTumIA  Raidionics | | 1.20  **1.07**  **1.07** | 1.04  **0.95**  **1.20** | 2.03  **2.17**  **2.13** | 2.10  **1.96**  **1.74** | 0.06  **0.006**  **0.03** | 0.94  **0.86**  **0.90** | 0.47  **0.44**  **0.38** | 1.24  **1.36**  **1.25** | 1.37  **1.24**  **1.15** | 0.12  **0.01**  **0.01** |
| **Necrosis** | **Min**  Manual  DeepBraTumIA  Raidionics | | 0.14  0.10  0.16 | 0.34  0.36  0.34 | 0.08  -0.07  0.002 | 0.42  0.54  0.37 | 0.36  0.12  0.21 | 0.09  0.07  0.12 | 0.32  0.16  0.35 | 0.02  -0.02  -0.01 | 0.30  0.49  0.20 | 0.49  0.10  0.10 |
|  | **10th percentile**  Manual  DeepBraTumIA  Raidionics | | 0.24  0.23  0.26 | 0.35  0.32  0.35 | 0.36  0.36  0.32 | 0.39  0.34  0.37 | 0.15  0.21  0.33 | 0.18  0.19  0.19 | 0.27  0.24  0.27 | 0.24  0.22  0.21 | 0.23  0.20  0.20 | 0.81  0.88  0.98 |
|  | **Median**  Manual  DeepBraTumIA  Raidionics | | 0.40  0.39  0.36 | 0.34  0.31  0.35 | 0.56  0.55  0.57 | 0.36  0.40  0.37 | 0.08  0.07  0.07 | 0.27  0.31  0.32 | 0.29  0.24  0.30 | 0.40  0.35  0.37 | 0.27  0.31  0.30 | 0.53  0.45  0.49 |
|  | **90th percentile**  Manual  DeepBraTumIA  Raidionics | | **0.65**  **0.59**  **0.48** | **0.45**  **0.30**  **0.39** | **1.01**  **0.90**  **0.86** | **0.49**  **0.65**  **0.56** | **0.04**  **0.03**  **0.03** | 0.46  0.45  0.40 | 0.37  0.32  0.28 | 0.69  0.67  0.55 | 0.35  0.42  0.42 | 0.25  0.21  0.19 |
|  | **Max**  Manual  DeepBraTumIA  Raidionics | | **0.90**  **0.86**  **0.69** | **0.68**  **0.81**  **0.61** | **1.50**  **1.43**  **1.37** | **1.16**  **1.16**  **1.12** | **0.02**  **0.03**  **0.02** | 0.72  0.78  **0.58** | 0.30  0.42  **0.20** | 0.98  0.98  **0.93** | 0.69  0.60  **0.54** | 0.10  0.10  **0.03** |
| **Edema** | **Min**  Manual  DeepBraTumIA  Raidionics | | -0.58  -0.47  -0.69 | 1.26  1.22  1.92 | -0.58  -0.57  -0.61 | 2.60  2.49  2.73 | 0.55  0.78  0.60 | -0.28  -0.27  -0.33 | 1.11  1.38  1.15 | -0.46  -0.22  -0.39 | 1.59  1.83  1.67 | 0.98  0.98  0.98 |
|  | **10th percentile**  Manual  DeepBraTumIA  Raidionics | | 0.26  0.22  0.25 | 0.24  0.26  0.27 | 0.29  0.28  0.28 | 0.24  0.30  0.30 | 0.39  0.39  0.39 | 0.24  0.28  0.25 | 0.20  0.22  0.20 | 0.25  0.22  0.25 | 0.23  0.24  0.23 | 0.98  0.98  0.98 |
|  | **Median**  Manual  DeepBraTumIA  Raidionics | | 0.56  0.52  0.54 | 0.35  0.33  0.38 | 0.62  0.61  0.60 | 0.29  0.33  0.36 | 0.25  0.22  0.22 | 0.50  0.52  0.52 | 0.22  0.22  0.21 | 0.51  0.51  0.50 | 0.31  0.30  0.29 | 0.98  0.98  0.98 |
|  | **90th percentile**  Manual  DeepBraTumIA  Raidionics | | 0.83  0.39  0.84 | 0.43  0.82  0.45 | 1.05  1.07  1.09 | 0.58  0.54  0.57 | 0.22  0.22  0.22 | 0.77  0.73  0.78 | 0.25  0.26  0.30 | 0.86  0.86  0.83 | 0.42  0.35  0.36 | 0.98  0.98  0.98 |
|  | **Max**  Manual  DeepBraTumIA  Raidionics | | 2.01  1.67  1.90 | 1.41  1.17  2.07 | 2.82  2.24  2.48 | 3.13  2.78  2.82 | 0.25  0.22  0.25 | 1.65  1.61  1.90 | 0.78  0.71  0.97 | 1.85  1.73  1.68 | 0.88  0.80  0.82 | 0.98  0.98  0.98 |
|  |  | | **APTw/CEST@2ppm ratio** | | | | |  |  |  |  |  |
|  |  | | **MGMTpm** | | **non-MGMTpm** | |  |  |  |  |  |  |
| **Segment** | **Parameter and method** | | **Median** | **IQR** | **Median** | **IQR** | **P-value** |  |  |  |  |  |
| **ET** | **Max**  Manual  DeepBraTumIA  Raidionics | | 3.46  3.28  3.37 | 6.47  7.13  8.97 | 8.22  15.86  23.22 | 52.65  66.85  78.56 | 0.22  0.22  0.22 |  |  |  |  |  |
| **Necrosis** | **Min**  Manual  DeepBraTumIA  Raidionics | | 0.43  0.49  0.51 | 7.24  6.27  3.78 | -2.19  -6.04  -3.67 | 20.72  28.93  15.01 | 0.33  0.09  0.11 |  |  |  |  |  |
|  | **Max**  Manual  DeepBraTumIA  Raidionics | | 2.24  2.32  **1.80** | 6.86  26.45  **1.35** | 4.17  8.69  **3.85** | 19.59  28.31  **9.57** | 0.29  0.09  **0.03** |  |  |  |  |  |
| **Edema** | **Median**  Manual  DeepBraTumIA  Raidionics | | 0.90  0.90  0.90 | 0.18  0.16  0.17 | 0.98  0.97  0.99 | 0.13  0.14  0.14 | 0.19  0.19  0.19 |  |  |  |  |  |

MGMT = O6-methylguanine-DNA methyltransferase. MGMTpm = MGMT promoter methylation. APTw = Amide proton transfer-weighted signal at 3.5ppm. CEST@2ppm = CEST signal at 2.0 ppm. IQR = Interquartile range. ET = Contrast-enhancing tumor.

**Supplementary Table 2.** Summary of the parameters that showed significant differences in the normalized APTw% (nAPTw%) and CEST@2ppm% (nCEST@2ppm%) in patients with glioblastoma (n = 39). P-values reflect the comparison between MGMT promoter methylated (MGMTpm) and non-methylated (non-MGMTpm) tumors. The data was nonparametric; therefore, the median values and interquartile range (IQR) are presented for each variable. P-values after correction for multiple comparisons are presented, with significant differences in bold.

|  |  | **nAPTw%** | | | | |
| --- | --- | --- | --- | --- | --- | --- |
| **Segment** | **Parameter and method** | **MGMTpm** | | **non-MGMTpm** | | **P-value** |
|  |  | **Median** | **IQR** | **Median** | **IQR** |  |
| **ET** | **Median**  Manual  DeepBraTumIA  Raidionics | 0.47  **0.45**  0.47 | 0.43  **0.41**  0.41 | 0.71  **0.71**  0.71 | 0.45  **0.40**  0.44 | 0.08  **0.04**  0.08 |
|  | **90th percentile**  Manual  DeepBraTumIA  Raidionics | 0.65  **0.65**  **0.67** | 0.73  **0.71**  **0.72** | 1.15  **1.17**  **1.14** | 0.54  **0.68**  **0.51** | 0.08  **0.03**  **0.04** |
|  | **Max**  Manual  DeepBraTumIA  Raidionics | **1.22**  **1.14**  **1.07** | **1.03**  **0.95**  **1.19** | **2.17**  **2.18**  **2.15** | **2.17**  **1.96**  **2.00** | **0.03**  **0.008**  **0.02** |
| **Necrosis** | **Median**  Manual  DeepBraTumIA  Raidionics | 0.39  **0.38**  **0.33** | 0.38  **0.27**  **0.34** | 0.56  **0.59**  **0.60** | 0.36  **0.40**  **0.37** | 0.07  **0.03**  **0.03** |
|  | **90th percentile**  Manual  DeepBraTumIA  Raidionics | **0.64**  **0.55**  **0.48** | **0.49**  **0.29**  **0.45** | **1.01**  **0.91**  **0.88** | **0.49**  **0.60**  **0.54** | **0.047**  **0.01**  **0.02** |
|  | **Max**  Manual  DeepBraTumIA  Raidionics | **0.87**  **0.74**  **0.68** | **0.76**  **0.45**  **0.58** | **1.50**  **1.57**  **1.43** | **1.16**  **1.42**  **1.15** | **0.03**  **0.008**  **0.01** |
|  |  | **nCEST@2ppm%** | | | | |
| **Segment** | **Parameter and method** | **MGMTpm** | | **non-MGMTpm** | |  |
|  |  | **Median** | **IQR** | **Median** | **IQR** | **P-value** |
| **ET** | **Max**  Manual  DeepBraTumIA  Raidionics | 0.94  **0.91**  **0.92** | 0.41  **0.46**  **0.35** | 1.25  **1.38**  **1.36** | 1.34  **1.24**  **1.19** | 0.08  **0.02**  **0.02** |
| **Necrosis** | **Max**  Manual  DeepBraTumIA  Raidionics | 0.69  0.77  **0.56** | 0.32  0.35  **0.20** | 0.98  0.96  **0.92** | 0.69  0.75  **0.62** | 0.12  0.06  **0.02** |

MGMT = O6-methylguanine-DNA methyltransferase. MGMTpm = MGMT promoter methylation. APTw = Amide proton transfer-weighted signal at 3.5ppm. CEST@2ppm = CEST signal at 2.0 ppm. IQR = Interquartile range. ET = Contrast-enhancing tumor.

**Supplementary Table 3.** Summary of the Spearman correlation coefficients as a function of MGMT promoter methylation (MGMTpm) percentage and receiver operating characteristic (ROC) analyses in the normalized APTw% (nAPTw%), normalized CEST@2ppm% (nCEST@2ppm%) and APTw/CEST@2ppm ratio that showed significant differences between MGMTpm and non-MGMTpm tumors, in the whole cohort (n = 44). The optimal cut-off is based on Youden’s index. P-values after correction for multiple comparisons are presented, with significant differences in bold.

| **nAPTw%** | | | | | | | | | |
| --- | --- | --- | --- | --- | --- | --- | --- | --- | --- |
|  |  | **Spearman** | | **ROC** | | | | | |
| **Segment** | **Parameter and method** | **r_s_** | **P-value** | **AUC** | **95% CI** | **Cut-off** | **Sensitivity** | **Specificity** | **P-value** |
| **ET** | **90th percentile**  Manual  DeepBraTumIA  Raidionics | **-0.34**  **-0.43**  **-0.44** | **0.04**  **0.01**  **0.01** | **0.74**  **0.74**  **0.75** | **0.58-0.90**  **0.59-0.90**  **0.59-0.91** | **0.70**  **0.71**  **0.69** | **100%**  **100%**  **100%** | **55%**  **55%**  **55%** | **0.02**  **0.01**  **0.01** |
|  | **Max**  Manual  DeepBraTumIA  Raidionics | **-0.41**  **-0.47**  **-0.46** | **0.01**  **0.01**  **0.01** | **0.80**  **0.82**  **0.77** | **0.65-0.94**  **0.69-0.96**  **0.61-0.92** | **1.50**  **1.41**  **1.47** | **88%**  **94%**  **88%** | **73%**  **64%**  **68%** | **0.007**  **0.007**  **0.01** |
| **Necrosis** | **90th percentile**  Manual  DeepBraTumIA  Raidionics | **-0.39**  **-0.36**  **-0.39** | **0.02**  **0.02**  **0.02** | **0.74**  **0.76**  **0.78** | **0.59-0.90**  **0.61-0.92**  **0.63-0.93** | **0.57**  **0.73**  **0.49** | **100%**  **81%**  **100%** | **46%**  **73%**  **55%** | **0.01**  **0.01**  **0.009** |
|  | **Max**  Manual  DeepBraTumIA  Raidionics | **-0.45**  **-0.34**  **-0.42** | **0.01**  **0.03**  **0.01** | **0.81**  **0.76**  **0.81** | **0.67-0.95**  **0.61-0.92**  **0.67-0.95** | **1.13**  **1.16**  **1.20** | **88%**  **81%**  **75%** | **73%**  **73%**  **82%** | **0.007**  **0.01**  **0.007** |
| **nCEST@2ppm%** | | | | | | | | | |
|  |  | **Spearman** | | **ROC** | | | | | |
| **Segment** | **Parameter and method** | **r_s_** | **P-value** | **AUC** | **95% CI** | **Cut-off** | **Sensitivity** | **Specificity** | **P-value** |
| **ET** | **Max**  Manual  DeepBraTumIA  Raidionics | **-0.35**  **-0.44**  **-0.47** | **0.03**  **0.01**  **0.01** | **0.78**  **0.85**  **0.80** | **0.63-0.93**  **0.73-0.97**  **0.66-0.94** | **1.03**  **0.96**  **0.93** | **88%**  **100%**  **100%** | **68%**  **64%**  **59%** | **0.009**  **0.005**  **0.007** |
| **Necrosis** | **Max**  Manual  DeepBraTumIA  Raidionics | **-0.34**  -0.27  **-0.45** | **0.04**  0.08  **0.01** | **0.74**  0.67  **0.78** | **0.58-0.91**  0.49-0.84  **0.64-0.93** | **0.81**  0.91  **0.73** | **81%**  69%  **75%** | **64%**  68%  **82%** | **0.01**  0.09  **0.009** |
| **APTw/CEST@2ppm ratio** | | | | | | | | | |
|  |  | **Spearman** | | **ROC** | | | | | |
| **Segment** | **Parameter and method** | **r_s_** | **P-value** | **AUC** | **95% CI** | **Cut-off** | **Sensitivity** | **Specificity** | **P-value** |
| **Necrosis** | **Max**  Manual  DeepBraTumIA  Raidionics | -0.26  **-0.40**  **-0.48** | 0.11  **0.02**  **0.01** | 0.63  0.67  **0.76** | 0.45-0.82  0.50-0.85  **0.59-0.92** | 3.21  2.51  **2.49** | 69%  94%  **88%** | 68%  50%  **68%** | 0.16  0.08  **0.01** |

MGMT = O6-methylguanine-DNA methyltransferase. MGMTpm = MGMT promoter methylation. APTw = Amide proton transfer-weighted signal at 3.5 ppm. CEST@2ppm = CEST signal at 2.0 ppm. ET = Contrast-enhancing tumor. ROC = Receiver operating characteristic. r_s_ = Spearman’s rank correlation coefficient. AUC = Area under curve. CI = Confidence interval.

**Supplementary Table 4.** Summary of the normalized APTw% (nAPTw%) and CEST@2ppm% (nCEST@2ppm%) values from the three segmentation methods comparing MGMT promoter methylated (MGMTpm) and non-methylated (non-MGMTpm) tumors in the whole cohort (n = 44). The data was nonparametric; therefore, the median values and interquartile range (IQR) are presented for each variable. Uncorrected p-values are presented, with significant differences are bold.

|  |  | **nAPTw%** | | | | | **nCEST@2ppm%** | | | | |
| --- | --- | --- | --- | --- | --- | --- | --- | --- | --- | --- | --- |
| **Segment** | **Parameter and method** | **MGMTpm** | | **non-MGMTpm** | |  | **MGMTpm** | | **non-MGMTpm** | |  |
|  |  | **Median** | **IQR** | **Median** | **IQR** | **P-value** | **Median** | **IQR** | **Median** | **IQR** | **P-value** |
| **ET** | **Min**  Manual  DeepBraTumIA  Raidionics | 0.04  0.10  0.06 | 0.31  0.40  0.29 | 0.10  -0.12  -0.09 | 0.55  0.82  0.61 | 0.51  0.053  0.19 | -0.001  0.04  0.04 | 0.43  0.36  0.39 | -0.02  -0.02  -0.14 | 0.37  0.47  0.46 | 0.45  0.20  0.06 |
|  | **10th percentile**  Manual  DeepBraTumIA  Raidionics | 0.30  0.29  0.29 | 0.36  0.36  0.32 | 0.40  0.40  0.43 | 0.34  0.36  0.37 | 0.19  0.37  0.26 | 0.26  0.26  0.24 | 0.31  0.29  0.30 | 0.22  0.22  0.20 | 0.26  0.27  0.27 | 0.76  1.00  0.94 |
|  | **Median**  Manual  DeepBraTumIA  Raidionics | 0.52  **0.47**  0.50 | 0.43  **0.42**  0.41 | 0.67  **0.71**  0.71 | 0.46  **0.44**  0.47 | 0.10  **0.04**  0.10 | 0.41  0.41  0.40 | 0.31  0.31  0.31 | 0.42  0.43  0.43 | 0.31  0.32  0.34 | 0.68  0.54  0.65 |
|  | **90th percentile**  Manual  DeepBraTumIA  Raidionics | 0.74  **0.74**  **0.75** | 0.64  **0.61**  **0.60** | 1.13  **1.17**  **1.13** | 0.56  **0.61**  **0.47** | 0.07  **0.01**  **0.01** | 0.61  **0.59**  0.62 | 0.35  **0.33**  0.31 | 0.78  **0.78**  0.80 | 0.52  **0.26**  0.28 | 0.29  **0.03**  0.06 |
|  | **Max**  Manual  DeepBraTumIA  Raidionics | **1.20**  **1.07**  **1.07** | **1.04**  **0.95**  **1.20** | **2.03**  **2.17**  **2.13** | **2.10**  **1.96**  **1.74** | **0.02**  **<0.001**  **0.003** | **0.94**  **0.86**  **0.90** | **0.47**  **0.44**  **0.38** | **1.24**  **1.36**  **1.25** | **1.37**  **1.24**  **1.15** | **0.03**  **<0.001**  **0.002** |
| **Necrosis** | **Min**  Manual  DeepBraTumIA  Raidionics | 0.14  0.10  0.16 | 0.34  0.36  0.34 | 0.08  -0.07  0.002 | 0.42  0.54  0.37 | 0.36  0.08  0.17 | 0.09  **0.07**  **0.12** | 0.32  **0.16**  **0.35** | 0.02  **-0.02**  **-0.01** | 0.30  **0.49**  **0.20** | 0.36  **0.03**  **0.02** |
|  | **10th percentile**  Manual  DeepBraTumIA  Raidionics | 0.24  0.23  0.26 | 0.35  0.32  0.35 | 0.36  0.36  0.32 | 0.39  0.34  0.37 | 0.11  0.18  0.31 | 0.18  0.19  0.19 | 0.27  0.24  0.27 | 0.24  0.22  0.21 | 0.23  0.20  0.20 | 0.71  0.83  0.98 |
|  | **Median**  Manual  DeepBraTumIA  Raidionics | **0.40**  **0.39**  **0.36** | **0.34**  **0.31**  **0.35** | **0.56**  **0.55**  **0.57** | **0.36**  **0.40**  **0.37** | **0.045**  **0.04**  **0.03** | 0.27  0.31  0.32 | 0.29  0.24  0.30 | 0.40  0.35  0.37 | 0.27  0.31  0.30 | 0.42  0.27  0.33 |
|  | **90th percentile**  Manual  DeepBraTumIA  Raidionics | **0.65**  **0.59**  **0.48** | **0.45**  **0.30**  **0.39** | **1.01**  **0.90**  **0.86** | **0.49**  **0.65**  **0.56** | **0.02**  **0.01**  **0.01** | 0.46  0.45  0.40 | 0.37  0.32  0.28 | 0.69  0.67  0.55 | 0.35  0.42  0.42 | 0.13  0.10  0.07 |
|  | **Max**  Manual  DeepBraTumIA  Raidionics | **0.90**  **0.86**  **0.69** | **0.68**  **0.81**  **0.61** | **1.50**  **1.43**  **1.37** | **1.16**  **1.16**  **1.12** | **0.003**  **0.01**  **0.001** | **0.72**  **0.78**  **0.58** | **0.30**  **0.42**  **0.20** | **0.98**  **0.98**  **0.93** | **0.69**  **0.60**  **0.54** | **0.03**  **0.03**  **0.002** |
| **Edema** | **Min**  Manual  DeepBraTumIA  Raidionics | -0.58  -0.47  -0.69 | 1.26  1.22  1.92 | -0.58  -0.57  -0.61 | 2.60  2.49  2.73 | 0.48  0.78  0.56 | -0.28  -0.27  -0.33 | 1.11  1.38  1.15 | -0.46  -0.22  -0.39 | 1.59  1.83  1.67 | 0.40  0.80  0.51 |
|  | **10th percentile**  Manual  DeepBraTumIA  Raidionics | 0.26  0.22  0.25 | 0.24  0.26  0.27 | 0.29  0.28  0.28 | 0.24  0.30  0.30 | 0.30  0.30  0.31 | 0.24  0.28  0.25 | 0.20  0.22  0.20 | 0.25  0.22  0.25 | 0.23  0.24  0.23 | 0.96  0.59  0.92 |
|  | **Median**  Manual  DeepBraTumIA  Raidionics | 0.56  0.52  0.54 | 0.35  0.33  0.38 | 0.62  0.61  0.60 | 0.29  0.33  0.36 | 0.15  0.06  0.09 | 0.50  0.52  0.52 | 0.22  0.22  0.21 | 0.51  0.51  0.50 | 0.31  0.30  0.29 | 0.65  0.85  0.98 |
|  | **90th percentile**  Manual  DeepBraTumIA  Raidionics | **0.83**  **0.39**  **0.84** | **0.43**  **0.82**  **0.45** | **1.05**  **1.07**  **1.09** | **0.58**  **0.54**  **0.57** | **0.03**  **0.03**  **0.045** | 0.77  0.73  0.78 | 0.25  0.26  0.30 | 0.86  0.86  0.83 | 0.42  0.35  0.36 | 0.33  0.38  0.52 |
|  | **Max**  Manual  DeepBraTumIA  Raidionics | 2.01  1.67  1.90 | 1.41  1.17  2.07 | 2.82  2.24  2.48 | 3.13  2.78  2.82 | 0.12  0.08  0.14 | 1.65  1.61  1.90 | 0.78  0.71  0.97 | 1.85  1.73  1.68 | 0.88  0.80  0.82 | 0.27  0.37  0.87 |

MGMT = O6-methylguanine-DNA methyltransferase. MGMTpm = MGMT promoter methylation. APTw = Amide proton transfer-weighted signal at 3.5ppm. CEST@2ppm = CEST signal at 2.0 ppm. IQR = Interquartile range. ET = Contrast-enhancing tumor.

**Supplementary Table 5.** Summary of the parameters that showed significant differences in the amide/CEST@2ppm ratio in the whole cohort (n = 44). P-values reflect the comparison between MGMT promoter methylated (MGMTpm) and non-methylated (non-MGMTpm) tumors. The data was nonparametric; therefore, the median values and interquartile range (IQR) are presented for each variable. Uncorrected p-values are presented, with significant differences in bold.

| **APTw/CEST@2ppm ratio** | | | | | | |
| --- | --- | --- | --- | --- | --- | --- |
| **Segment** | **Parameter and method** | **MGMTpm** | | **non-MGMTpm** | |  |
|  |  | **Median** | **IQR** | **Median** | **IQR** | **P-value** |
| **ET** | **Max**  Manual  DeepBraTumIA  Raidionics | 3.46  **3.28**  **3.37** | 6.47  **7.13**  **8.97** | 8.22  **15.86**  **23.22** | 52.65  **66.85**  **78.56** | 0.07  **0.04**  **0.02** |
| **Necrosis** | **Min**  Manual  DeepBraTumIA  Raidionics | 0.43  **0.49**  **0.51** | 7.24  **6.27**  **3.78** | -2.19  **-6.04**  **-3.67** | 20.72  **28.93**  **15.01** | 0.27  **0.02**  **0.03** |
|  | **Max**  Manual  DeepBraTumIA  Raidionics | 2.24  **2.32**  **1.80** | 6.86  **26.45**  **1.35** | 4.17  **8.69**  **3.85** | 19.59  **28.31**  **9.57** | 0.21  **0.02**  **0.002** |
| **Edema** | **Median**  Manual  DeepBraTumIA  Raidionics | **0.90**  **0.90**  **0.90** | **0.18**  **0.16**  **0.17** | **0.98**  **0.97**  **0.99** | **0.13**  **0.14**  **0.14** | **0.04**  **0.03**  **0.02** |

APTw = APTw signal at 3.5 ppm. CEST@2ppm = CEST signal at 2.0 ppm. MGMT = O6-methylguanine-DNA methyltransferase. MGMTpm = MGMT promoter methylation. IQR = Interquartile range. ET = Contrast-enhancing tumor.

**Supplementary Table 6.** Summary of the parameters that showed significant differences in the normalized APTw% (nAPTw%), normalized CEST@2ppm% (nCEST@2ppm%) and the APTw/CEST@2ppm ratio in patients with glioblastoma (n = 39). P-values reflect the comparison between MGMT promoter methylated (MGMTpm) and non-methylated (non-MGMTpm) tumors. The data was nonparametric; therefore, the median values and interquartile range (IQR) are presented for each variable. Uncorrected p-values are presented, with significant differences in bold.

|  |  | **nAPTw%** | | | | |
| --- | --- | --- | --- | --- | --- | --- |
| **Segment** | **Parameter and method** | **MGMTpm** | | **non-MGMTpm** | | **P-value** |
|  |  | **Median** | **IQR** | **Median** | **IQR** |  |
| **ET** | **Median**  Manual  DeepBraTumIA  Raidionics | **0.47**  **0.45**  **0.47** | **0.43**  **0.41**  **0.41** | **0.71**  **0.71**  **0.71** | **0.45**  **0.40**  **0.44** | **0.045**  **0.01**  **0.046** |
|  | **90th percentile**  Manual  DeepBraTumIA  Raidionics | **0.65**  **0.65**  **0.67** | **0.73**  **0.71**  **0.72** | **1.15**  **1.17**  **1.14** | **0.54**  **0.68**  **0.51** | **0.04**  **0.009**  **0.01** |
|  | **Max**  Manual  DeepBraTumIA  Raidionics | **1.22**  **1.14**  **1.07** | **1.03**  **0.95**  **1.19** | **2.17**  **2.18**  **2.15** | **2.17**  **1.96**  **2.00** | **0.007**  **<0.001**  **0.003** |
| **Necrosis** | **Min**  Manual  DeepBraTumIA  Raidionics | 0.12  **0.12**  0.15 | 0.37  **0.36**  0.38 | 0.08  **-0.11**  0.02 | 0.42  **0.58**  0.43 | 0.55  **0.048**  0.28 |
|  | **Median**  Manual  DeepBraTumIA  Raidionics | **0.39**  **0.38**  **0.33** | **0.38**  **0.27**  **0.34** | **0.56**  **0.59**  **0.60** | **0.36**  **0.40**  **0.37** | **0.045**  **0.01**  **0.01** |
|  | **90th percentile**  Manual  DeepBraTumIA  Raidionics | **0.64**  **0.55**  **0.48** | **0.49**  **0.29**  **0.45** | **1.01**  **0.91**  **0.88** | **0.49**  **0.60**  **0.54** | **0.03**  **0.002**  **0.004** |
|  | **Max**  Manual  DeepBraTumIA  Raidionics | **0.87**  **0.74**  **0.68** | **0.76**  **0.45**  **0.58** | **1.50**  **1.57**  **1.43** | **1.16**  **1.42**  **1.15** | **0.009**  **<0.001**  **0.002** |
| **Edema** | **Median**  Manual  DeepBraTumIA  Raidionics | 0.53  **0.48**  **0.51** | 0.38  **0.38**  **0.36** | 0.65  **0.62**  **0.62** | 0.27  **0.29**  **0.34** | 0.07  **0.03**  **0.03** |
|  | **90th percentile**  Manual  DeepBraTumIA  Raidionics | **0.79**  **0.76**  **0.80** | **0.46**  **0.46**  **0.41** | **1.06**  **1.07**  **1.09** | **0.56**  **0.51**  **0.62** | **0.02**  **0.01**  **0.02** |
|  | **Max**  Manual  DeepBraTumIA  Raidionics | **1.82**  **1.62**  **1.62** | **0.87**  **0.96**  **1.75** | **2.87**  **2.28**  **2.53** | **3.15**  **2.96**  **2.59** | **0.02**  **0.02**  **0.02** |
|  |  | **nCEST@2ppm%** | | | | |
| **Segment** | **Parameter and method** | **MGMTpm** | | **non-MGMTpm** | |  |
|  |  | **Median** | **IQR** | **Median** | **IQR** | **P-value** |
| **ET** | **90th percentile**  Manual  DeepBraTumIA  Raidionics | 0.58  **0.57**  0.60 | 0.37  **0.36**  0.33 | 0.80  **0.78**  0.81 | 0.43  **0.27**  0.34 | 0.22  **0.04**  0.12 |
|  | **Max**  Manual  DeepBraTumIA  Raidionics | **0.94**  **0.91**  **0.92** | **0.41**  **0.46**  **0.35** | **1.25**  **1.38**  **1.36** | **1.34**  **1.24**  **1.19** | **0.02**  **0.001**  **0.002** |
| **Necrosis** | **Min**  Manual  DeepBraTumIA  Raidionics | 0.07  **0.09**  0.12 | 0.34  **0.32**  0.33 | 0.02  **-0.04**  -0.01 | 0.30  **0.51**  0.22 | 0.55  **0.03**  0.07 |
|  | **90th percentile**  Manual  DeepBraTumIA  Raidionics | 0.44  **0.41**  **0.39** | 0.39  **0.24**  **0.24** | 0.69  **0.68**  **0.57** | 0.35  **0.38**  **0.40** | 0.13  **0.03**  **0.04** |
|  | **Max**  Manual  DeepBraTumIA  Raidionics | **0.69**  **0.77**  **0.56** | **0.32**  **0.35**  **0.20** | **0.98**  **0.96**  **0.92** | **0.69**  **0.75**  **0.62** | **0.049**  **0.008**  **0.001** |
|  |  | **APTw/CEST@2ppm ratio** | | | | |
| **Segment** | **Parameter and method** | **MGMTpm** | | **non-MGMTpm** | |  |
|  |  | **Median** | **IQR** | **Median** | **IQR** | **P-value** |
| **ET** | **Max**  Manual  DeepBraTumIA  Raidionics | 3.55  3.51  **3.86** | 6.31  6.91  **8.81** | 9.60  19.55  **29.25** | 61.36  70.74  **77.88** | 0.08  0.052  **0.01** |
| **Necrosis** | **Min**  Manual  DeepBraTumIA  Raidionics | 0.31  **0.53**  0.66 | 7.34  **3.07**  3.94 | -2.19  **-6.39**  -4.30 | 20.72  **29.60**  20.25 | 0.43  **0.008**  0.06 |
|  | **Max**  Manual  DeepBraTumIA  Raidionics | 2.48  **2.32**  **1.88** | 15.09  **25.36**  **3.43** | 4.17  **10.27**  **4.17** | 19.59  **30.89**  **9.96** | 0.42  **0.02**  **0.007** |
| **Edema** | **Median**  Manual  DeepBraTumIA  Raidionics | 0.90  0.92  **0.90** | 0.16  0.15  **0.16** | 0.98  0.97  **1.00** | 0.13  0.15  **0.15** | 0.06  0.07  **0.03** |

MGMT = O6-methylguanine-DNA methyltransferase. MGMTpm = MGMT promoter methylation. APTw = Amide proton transfer-weighted signal at 3.5ppm. CEST@2ppm = CEST signal at 2.0 ppm. IQR = Interquartile range. ET = Contrast-enhancing tumor.

**Supplementary Table 7.** Summary of the Spearman correlation coefficients as a function of MGMT promoter methylation (MGMTpm) percentage and receiver operating characteristic (ROC) analyses in the normalized APTw% (nAPTw%), normalized CEST@2ppm% (nCEST@2ppm%) and APTw/CEST@2ppm ratio that showed significant differences between MGMTpm and non-MGMTpm tumors, in the whole cohort (n = 44). The optimal cut-off is based on Youden’s index. Uncorrected p-values are presented, with significant differences in bold.

| **nAPTw%** | | | | | | | | | |
| --- | --- | --- | --- | --- | --- | --- | --- | --- | --- |
|  |  | **Spearman** | | **ROC** | | | | | |
| **Segment** | **Parameter and method** | **r_s_** | **P-value** | **AUC** | **95% CI** | **Cut-off** | **Sensitivity** | **Specificity** | **P-value** |
| **ET** | **Median**  Manual  DeepBraTumIA  Raidionics | -0.28  **-0.36**  -0.31 | 0.08  **0.02**  0.051 | **0.71**  **0.72**  **0.70** | **0.55-0.88**  **0.56-0.89**  **0.53-0.87** | **0.46**  **0.47**  **0.48** | **100%**  **94%**  **94%** | **50%**  **59%**  **55%** | **0.03**  **0.02**  **0.04** |
|  | **90th percentile**  Manual  DeepBraTumIA  Raidionics | **-0.34**  **-0.43**  **-0.44** | **0.03**  **0.005**  **0.004** | **0.74**  **0.74**  **0.75** | **0.58-0.90**  **0.59-0.90**  **0.59-0.91** | **0.70**  **0.71**  **0.69** | **100%**  **100%**  **100%** | **55%**  **55%**  **55%** | **0.01**  **0.01**  **0.01** |
|  | **Max**  Manual  DeepBraTumIA  Raidionics | **-0.41**  **-0.47**  **-0.46** | **0.007**  **0.002**  **0.003** | **0.80**  **0.82**  **0.77** | **0.65-0.94**  **0.69-0.96**  **0.61-0.92** | **1.50**  **1.41**  **1.47** | **88%**  **94%**  **88%** | **73%**  **64%**  **68%** | **0.002**  **0.001**  **0.005** |
| **Necrosis** | **Median**  Manual  DeepBraTumIA  Raidionics | **-0.32**  **-0.31**  **-0.34** | **0.047**  **0.04**  **0.03** | **0.70**  **0.72**  **0.73** | **0.53-0.87**  **0.55-0.89**  **0.57-0.89** | **0.61**  **0.44**  **0.37** | **50%**  **75%**  **88%** | **86%**  **64%**  **55%** | **0.04**  **0.02**  **0.02** |
|  | **90th percentile**  Manual  DeepBraTumIA  Raidionics | **-0.39**  **-0.36**  **-0.39** | **0.02**  **0.02**  **0.01** | **0.74**  **0.76**  **0.78** | **0.59-0.90**  **0.61-0.92**  **0.63-0.93** | **0.57**  **0.73**  **0.49** | **100%**  **81%**  **100%** | **46%**  **73%**  **55%** | **0.01**  **0.006**  **0.004** |
|  | **Max**  Manual  DeepBraTumIA  Raidionics | **-0.45**  **-0.34**  **-0.42** | **0.004**  **0.03**  **0.007** | **0.81**  **0.76**  **0.81** | **0.67-0.95**  **0.61-0.92**  **0.67-0.95** | **1.13**  **1.16**  **1.20** | **88%**  **81%**  **75%** | **73%**  **73%**  **82%** | **0.001**  **0.006**  **0.001** |
| **Edema** | **90th percentile**  Manual  DeepBraTumIA  Raidionics | -0.21  -0.27  -0.18 | 0.18  0.09  0.26 | 0.65  0.65  0.65 | 0.47-0.83  0.47-0.83  0.47-0.82 | 2.76  2.08  1.65 | 56%  63%  94% | 73%  64%  46% | 0.12  0.12  0.12 |
| **nCEST@2ppm%** | | | | | | | | | |
|  |  | **Spearman** | | **ROC** | | | | | |
| **Segment** | **Parameter and method** | **r_s_** | **P-value** | **AUC** | **95% CI** | **Cut-off** | **Sensitivity** | **Specificity** | **P-value** |
| **ET** | **90th percentile**  Manual  DeepBraTumIA  Raidionics | -0.21  **-0.37**  **-0.32** | 0.18  **0.02**  **0.045** | 0.66  **0.69**  0.66 | 0.48-0.84  **0.52-0.87**  0.48-0.84 | 0.70  **0.71**  0.69 | 75%  **75%**  75% | 68%  **68%**  64% | 0.10  **0.04**  0.09 |
|  | **Max**  Manual  DeepBraTumIA  Raidionics | **-0.35**  **-0.44**  **-0.47** | **0.03**  **0.004**  **0.002** | **0.78**  **0.85**  **0.80** | **0.63-0.93**  **0.73-0.97**  **0.66-0.94** | **1.03**  **0.96**  **0.93** | **88%**  **100%**  **100%** | **68%**  **64%**  **59%** | **0.004**  **<0.001**  **0.002** |
| **Necrosis** | **Min**  Manual  DeepBraTumIA  Raidionics | 0.22  0.29  **0.35** | 0.19  0.06  **0.03** | 0.61  0.65  0.68 | 0.42-0.79  0.48-0.83  0.51-0.86 | 0.07  0.08  0.06 | 59%  46%  68% | 69%  88%  69% | 0.26  0.11  0.055 |
|  | **Max**  Manual  DeepBraTumIA  Raidionics | **-0.34**  -0.27  **-0.45** | **0.04**  0.08  **0.004** | **0.74**  0.67  **0.78** | **0.58-0.91**  0.49-0.84  **0.64-0.93** | **0.81**  0.91  **0.73** | **81%**  69%  **75%** | **64%**  68%  **82%** | **0.01**  0.08  **0.003** |
| **APTw/CEST@2ppm ratio** | | | | | | | | | |
|  |  | **Spearman** | | **ROC** | | | | | |
| **Segment** | **Parameter and method** | **r_s_** | **P-value** | **AUC** | **95% CI** | **Cut-off** | **Sensitivity** | **Specificity** | **P-value** |
| **ET** | **Max**  Manual  DeepBraTumIA  Raidionics | -0.30  -0.28  **-0.42** | 0.054  0.08  **0.007** | **0.70**  **0.72**  **0.73** | **0.53-0.87**  **0.56-0.89**  **0.56-0.90** | **18.38**  **18.38**  **16.82** | **50%**  **56%**  **69%** | **91%**  **86%**  **82%** | **0.04**  **0.02**  **0.02** |
| **Necrosis** | **Min**  Manual  DeepBraTumIA  Raidionics | 0.19  0.28  0.29 | 0.24  0.07  0.07 | 0.64  0.69  0.68 | 0.46-0.82  0.51-0.86  0.50-0.85 | 0.58  -2.75  0.39 | 41%  73%  64% | 88%  69%  75% | 0.15  0.051  0.07 |
|  | **Max**  Manual  DeepBraTumIA  Raidionics | -0.26  **-0.40**  **-0.48** | 0.11  **0.01**  **0.002** | 0.63  0.67  **0.76** | 0.45-0.82  0.50-0.85  **0.59-0.92** | 3.21  2.51  **2.49** | 69%  94%  **88%** | 68%  50%  **68%** | 0.16  0.07  **0.008** |
| **Edema** | **Median**  Manual  DeepBraTumIA  Raidionics | **-0.32**  **-0.33**  **-0.35** | **0.04**  **0.03**  **0.02** | 0.67  0.67  **0.69** | 0.50-0.84  0.50-0.84  **0.52-0.86** | 0.91  0.91  **0.94** | 75%  81%  **69%** | 59%  55%  **68%** | 0.08  0.07  **0.048** |

MGMT = O6-methylguanine-DNA methyltransferase. MGMTpm = MGMT promoter methylation. APTw = Amide proton transfer-weighted signal at 3.5 ppm. CEST@2ppm = CEST signal at 2.0 ppm. ET = Contrast-enhancing tumor. ROC = Receiver operating characteristic. r_s_ = Spearman’s rank correlation coefficient. AUC = Area under curve. CI = Confidence interval.
